# Perception of Medical Professionalism among Medical Residents in Spain

**DOI:** 10.3390/healthcare9111580

**Published:** 2021-11-18

**Authors:** Joaquín García-Estañ, Jose María Cabrera-Maqueda, Eduardo González-Lozano, Jacinto Fernández-Pardo, Noemí M. Atucha

**Affiliations:** 1 Centro de Estudios en Educación Médica, Universidad de Murcia, 30120 Murcia, Spain; ntma@um.es; 2 Servicio de Docencia y Formación, Hospital Clínico Universitario Virgen de la Arrixaca, 30120 Murcia, Spain; josemaria.olvera@gmail.com (J.M.C.-M.); eduardo220590@gmail.com (E.G.-L.); 3 Comisión de Docencia, Hospital General Universitario Reina Sofía, 30120 Murcia, Spain; jafepa@um.es

**Keywords:** medical education, medical ethics, medical professionalism, medicine students, education in professionalism

## Abstract

Background: Medical professionalism, defined as commitment to the primacy of patient welfare, is the basis for doctor–patient–society relationships, but previous research with medical students has shown that professionalism and social commitment to medicine may be waning. To determine if this trend also appears in recently qualified practicing doctors, we surveyed 90 newly graduated doctors currently working as medical residents in two university hospitals in Murcia, Spain. A previously validated questionnaire that studies the perception of six categories (responsibility, altruism, service, excellence, honesty and integrity, and respect) defining medical professionalism was used. Results: A good perception of professionalism was found among medical residents, with more than 70% positive responses in all these six categories. There is an increasing trend in the number of negative responses as the residency goes on. Altruism was the category with the greatest percentage of negative answers (22.3%) and Respect was the category with the lowest percentage (12.9%). Conclusions: The results show a good professionalism perception in medical residents, but also a slight decline in positive answers that began during medical school. A significant trend was found when including both students and residents. Although there were some differences between students and residents, these were not statistically significant. Educational interventions are needed both at the level of medical school and postgraduate medical residency.

## 1. Introduction

The interest in medical professionalism, defined as a set of values, behaviors and relationships that underpin the trust that the public places in the doctors, has been growing in recent years, due to many reports [1,2,3,4,5,6,7,8,9] alerting about the lack of social commitment in a profession, medicine, that it is much more than a mere occupation. The rapid advancement of medical knowledge has had a great impact in medicine, and it has been suggested that the increase in professional expertise has been associated with a decrease in professionalism along with social commitment [1,2]. Social commitment encompasses a commitment to the patient, to fellow professionals, and to the institution or system within which healthcare is provided [10]. Medical practice relies on professionalism, since it is the base of the doctors’ relationship with patients and with society. As it has been shown [3,4,5,6], if well used, professionalism improves the doctor–patient–society relationships and increases patient satisfaction as well as satisfaction in healthcare professionals, thus making healthcare more effective and efficient.

Few studies in young interns or in residents can be found, but some showed an alarmingly low level of professionalism, indicating a 50% lack of professionalism competency [7,8]. Previously, a study analyzed the perception of medical professionalism in medical students of a medical school in Spain [9]. In this study, although the perception of professionalism was acceptable, a trend towards a slow decline during the medical school period was observed [9]. Thus, having seen this issue in a medical student cohort, this paper seeks to examine whether or not these trends persist in the same cohort once they become practicing residents or if these trends found among students are also found amongst residents.

These studies, the one performed in medical students [9] and the present one, are two completely different studies, both performed and analyzed with the same methodology (scale and statistics) but at different times, with a year of difference; one was conducted in February 2019 in medical students and the other in February 2020 in medical residents. Although the data on medical students are published [9], we have also compared the present data obtained in medical residents with those obtained previously in medical students, treating them effectively as a single cohort of individuals. This is because of the nature of the medical school–residency progression pattern in Spain. Thus, medical training in Spain follows the so-called Bologna Scheme, in which students undertake six years of theoretical and practical training in a medical school. Then, they enter medical specialization in hospitals and primary care centers, through a residency program (mainly of a public nature), where young doctors enter after they pass a national exam that allows them to select a desired specialty according to their position in the exam [11]. This training system therefore supports the argument to consider both medical students and residents as the same cohort.

## 2. Materials and Methods

This work was approved by the Research Ethics Committee of the University of Murcia (2282/2019) and by the Teaching Committees of the Virgen de la Arrixaca and Reina Sofía, both University hospitals, pertaining to the regional Health Service of Murcia, Spain. We used the same methodology as in our previous paper about medical students [9]. Briefly, we used the Professionalism questionnaire from the Penn State University School of Medicine (PSCOM), adapted to the Spanish language by Bustamante and Sanabria [12], with minimal changes to adapt it to Castilian Spanish. It consists of 6 blocks, each of which presents 6 attitudes that represent an element of medical professionalism defined by the American Board of Internal Medicine, namely: responsibility, altruism, service, excellence, honesty and integrity, and respect [13]. Firstly, the respondents were asked to order the attitudes according to the frequency of their compliance with these attitudes (5-point Likert scale: “Never, Little, Sometimes, Frequently and Always”). Secondly, the respondents were asked to rank the attitudes in order of importance (1 is considered the most important attitude of the block and 6 the least important). The survey was conducted online in February–March 2020 through a Google form sent by email to all residents. Participation was voluntary and anonymous. The number of residents participating was 90 out of a total of 325 (27.7%), 54.4% women and 45.6% men. Residents were from two university hospitals in Murcia, 72 from Hospital Clínico Universitario Virgen de la Arrixaca (out of 253) and 18 from Hospital General Universitario Reina Sofía (out of 72). This response rate is comparable to that observed in previous international studies [14,15].

Statistical analysis was performed with SPSS v24 (IBM, Armonk, NY, USA). In summary, the answers given to the different questions were converted to numerical data to obtain descriptive statistics. Since the data did not follow a normal distribution, the vast majority of the statistics were carried out with non-parametric tests [16]. To compare the categories between the courses, the Kruskal–Wallis test was used. Additionally, to compare the categories within each course, we used the Friedman test. In the same way, the Mann–Whitney test was used to compare categories between sexes. Finally, the data were grouped into negative and positive responses, assigning the responses “never”, “little” and “sometimes” as negative and the responses “frequently” and “always” as positive.

## 3. Results

**Figure 1:**
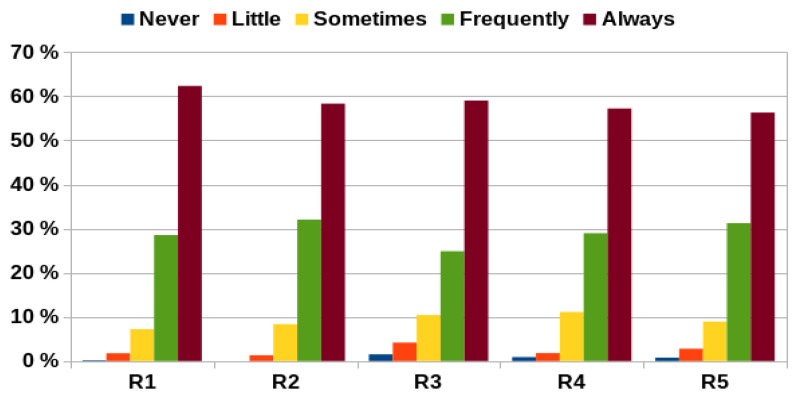
Percentage of responses based on year of residency (R, residency).

Cronbach’s alpha for each category was greater than 0.70, indicating an acceptable internal consistency and reliability of the survey. The mean alpha was 0.74 + 0.09. Figure 1 represents the responses obtained in the survey based on year of residency. Most of them were in the upper two grades (always and frequently) and no gender differences were found.

**Figure 2:**
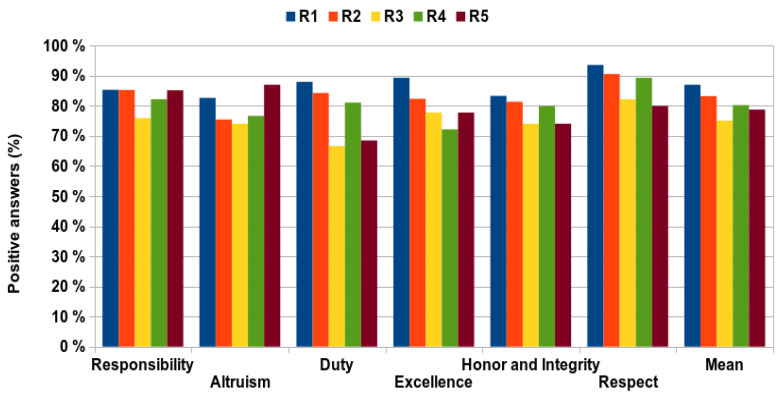
Mean percentage of professionalism categories in every year of residence.

**Figure 3:**
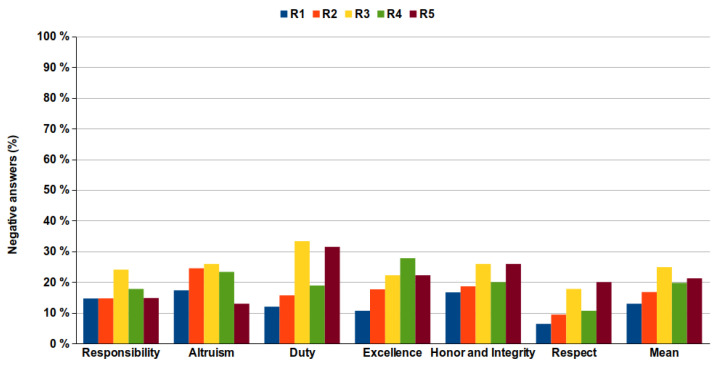
Percentage of negative responses by category and year of residence.

As shown in Figure 2, the percentage of positive responses obtained in relation to the maximum possible (100%) was quite high, practically 70% upwards in all categories. Figure 3 shows the percentage of negative responses obtained based on category and year of residency. An increasing trend in the number of negative responses can be observed as the residence advances with a maximum showed in the 3rd year. Altruism was the category with the highest percentage of negative answers (22.0%) and Respect was the category with the lowest percentage (12.9%). On the other hand, the first year was the one with the least negative responses (12.9%) and the third year showed the greatest percentage (24.9%), with a mean across the five years of 19.1%.

**Figure 4:**
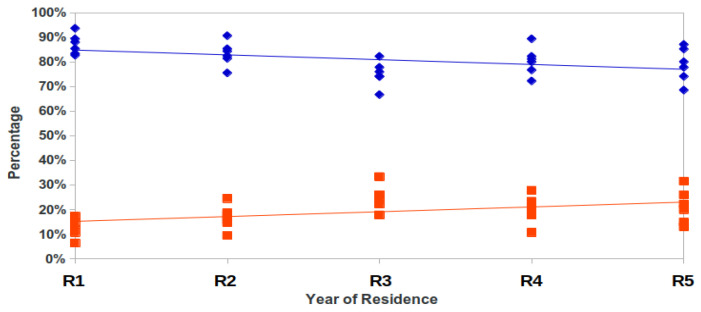
Trendline of positive (blue) and negative (red) answers in medical residents (R, resident).

Both negative and positive responses were significantly related (Kruskal–Wallis test, *p* = 0.013) (Figure 4) with the year of residency (negative responses increase along with courses and positive responses decrease), which confirms the slight tendency that negative responses increase along with course, as it can be observed in Figure 2 and Figure 3.

**Figure 5:**
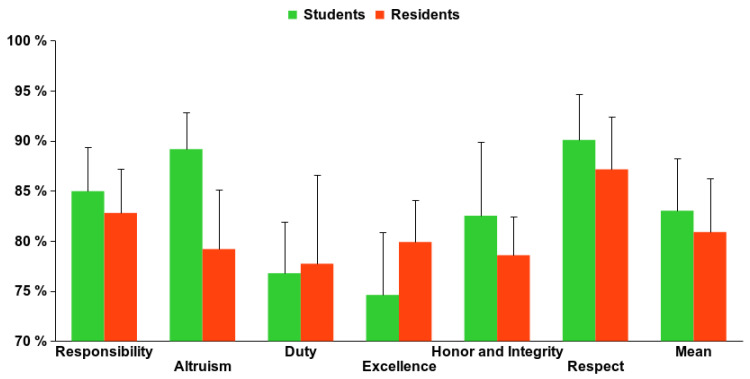
Comparison between positive answers in Residents and Medical Students.

**Figure 6:**
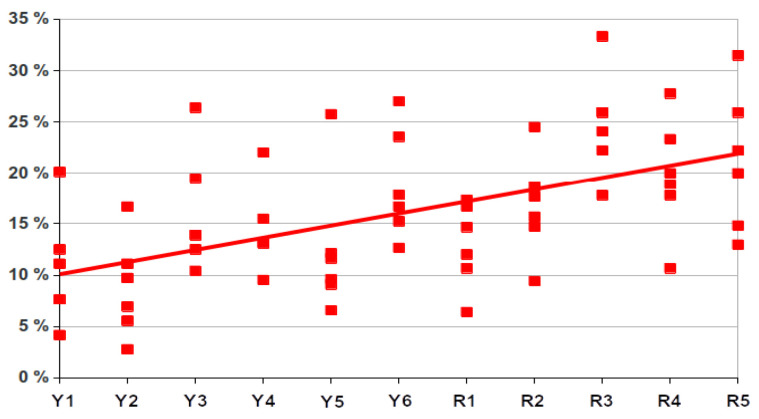
Trendline with data on residents (R1 to R5) and data on medical students (Y1 to Y6) of our previous study [9].

In this regard, in order to compare the data presented here and those obtained in our previous paper in medical students [9], we have prepared two figures showing the comparison of positive answers in every category (Figure 5) and the trend that can be observed in negative answers both during medical school time and during residency (Figure 6). In fact, the residents in their first year are those that graduated one year after we performed the study on medical students; thus, that first year of residency could be considered as the seventh year after having started medical school. As observed in Figure 5, there were some differences between students and residents, but they were not significant. The Kruskal–Wallis test also show a significant relationship with both students and residents (Figure 6).

**Figure 7:**
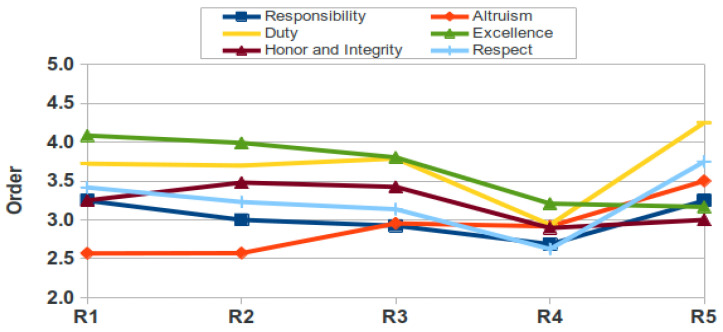
Order of importance of categories for female residents (R, resident).

**Figure 8:**
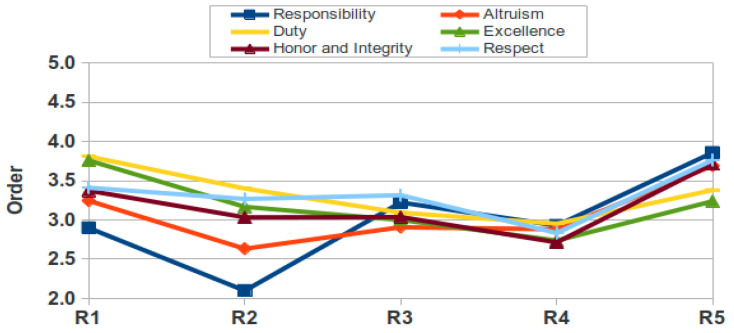
Order of importance of categories for male residents (R, resident).

Figure 7 and Figure 8 show the different categories in order of importance according to residents’ ranking. In men, the domain best considered was Responsibility (mean of 3.0 in the 5 years), whereas in women it was Altruism (mean 2.9), while the least considered was Duty in both men and women (means of 3.3 and 3.7, resp.). Interestingly, these figures show in both genders an increase in the last year (R5) in most of the six elements. In men, four of these R5 values are the highest (responsibility, altruism, honor and integrity and respect) of all years whereas in women, five out of six are the highest ever (the same four as in men plus duty).

Finally, Table 1 shows that the mean of positive answers was slightly lower than those obtained in the study on medical students [9], but this does not reach statistical significance.

## 4. Discussion

In a previous paper [9], we analyzed the perception of professionalism in medical students from our university. Generally speaking, the perception was good, showing more than 75% of positive answers in all six categories or attitudes. In the present paper, the perception was analyzed in medical residents of two university hospitals in our city, where most of our medical students decided to choose the medical specialization residency. Since the surveys were carried out sequentially, we believe that these results could be thought of as a single cohort of young medical doctors when they started medical school (surveyed in 2019, they will enter residency in 2021), and continuing with the residency period until they obtain their medical specialization (surveyed in 2020, R1 graduated in 2018). However, some uncertainties persist since medical students and residents are not directly comparable, mainly due to the changes in responsibility and stress, among other factors [17].

Regarding the data on residents, our results show that there is a good perception in all attitudes since all of them had positive answers greater than 70% on average, but a little lower than observed previously [9]. The best-rated category was Respect, with 87.2% of positive answers, which is slightly lower than the data obtained in the medical students (Table 1). Our results are similar to those previously published showing good responses, but not very close to the maximum; in fact, the data are somewhat lower, with a 79.5% score [18]. Although there were no significant differences between students and residents, a lower number of positive answers is obtained in four out of six attitudes in residents, thus suggesting that the reduction in professional attitudes observed during the medical school period is maintained throughout the residency.

In previous articles [9,18], more negative responses were collected in the clinical courses of the degree compared to non-clinical courses. As observed in the present results, the trend is maintained and the percentage of negative responses continues to increase in the postgraduate years during the residency period. A negative trend in ethical progress during the Medicine studies has been previously published [19,20,21,22,23,24] and our data extend these observations to also include the residency years. Moreover, when the data of both studies are shown together (Figure 6), a clear trend to a significant increase in negative answers is shown. As observed, there is a doubling of negative answers after students enter medical school, indicating that the period of specialization does not improve professionalism attitudes. However, a word of caution is necessary since we only surveyed a year, and a longitudinal study with more years would be necessary to rule out that doctors become more professional with time.

Many authors have wondered about the causes of this decrease in ethical values during medical school and the medical specialization period. Among them are the loss of empathy over time, a desire to be less emotionally involved with patients, mental health issues as well as difficulties in patients’ communication and problems related to interpersonal relationships in the hospital [25,26,27]. Sometimes, both students and residents observe unethical behaviours in their tutors. In fact, medical residents seem to prefer teaching methods that stress the importance of faculty and colleague role-modelling, the culture of professionalism within their institutions, and the importance of evaluation and feedback [28,29,30,31,32]. All of these issues may be part of the hidden curriculum, which is implicitly taught by example day to day, not the explicit teaching of lectures and so on [33], which, as our findings seem to suggest, could well be the center of new curricular interventions in this area. However, this is outside the scope of the present work and would require further study.

The relative value assigned to different professional attitudes varied considerably, as occurred in medical students [9] with the values given for Altruism. The domains Altruism, Duty, Excellence and Honor and Integrity are valued below the mean. Although we cannot discriminate among medical specialities, it has been published that residents perceive differences in the relative importance of professional attitudes [34], which may be interesting when teaching professional behavior to address potential gaps. A study among residents revealed a conflict between self-interest and altruism and when they have to manage interpersonal problematic interactions [35]. It is important that both tutors and residents are aware of their behaviour and attitudes during the medical practice, especially when they interact with their colleagues, so that students’ professional behavior can be improved [36]. As teaching by example is identified as a common educational method, faculty must be aware of the role their behavior and attitudes have in shaping resident behavior and attitudes [37]. Another interesting result deserves a small commentary: the slight increase in the importance rank (Figure 7 and Figure 8) in most categories in residents of the last year (R5), which would be interesting to explore further, since it may suggest a drastic change once they know that their resident period is about to finish.

The scale used to assess professionalism, which measures perception, a qualitative not an actual quantitative measure, is one of the limitations of the study. Since participation was voluntary, those who responded may be more motivated than those who did not [19]. These two limitations, thus, would give more positive results of professionalism than the real ones. Another limitation is possible if the medical residents knew of our previous study in medical students, therefore that could have influenced their responses. This may be possible; however the results of the medical students were published [9] long after the resident survey was finished. Finally, it would be also interesting to obtain qualitative data to discover why those values decrease over time. Therefore, the faculty and tutors should be encouraged to make specific programs to improve professionalism among their students and residents, since a good doctor, to be a good professional, must not only have theoretical and practical knowledge, but also proper ethical values [38,39].

## 5. Conclusions

The results show a good professionalism perception in medical residents, which declines slightly as residency advances. A significant linear relationship was found when including both students and residents, indicating that the decline observed during medical residency is a continuation of what was observed during medical school. Educational interventions are needed both at the level of medical school and postgraduate medical residency. Improving professionalism in young doctors would be of benefit not only for patients’ healthcare but also for their satisfaction and motivation.

## Figures and Tables

**Table 1:** Average percentage of positive answers in residents compared with those obtained in a previous study in medical students [9].

Categories	Students	Residents
Responsibility	84.97% ± 0.04	82.79% ± 0.04
Altruism	89.17% ± 0.04	79.19% ± 0.05
Duty	76.77% ± 0.05	77.72% ± 0.08
Excellence	74.61% ± 0.06	79.89% ± 0.04
Honor and Integrity	82.51% ± 0.07	78.57% ± 0.03
Respect	90.08% ± 0.05	87.15% ± 0.05
Mean	83.02% ± 0.05	80.89% ± 0.05

## Data Availability

Data is available upon request. Data are in Spanish in an excel file. Please contact jgestan@um.es for details.
